# Preventing Impaired Driving Opportunities and Problems

**Published:** 2011

**Authors:** Robert B. Voas, James C. Fell

**Keywords:** Problematic alcohol use, prevention, alcohol-related injury prevention, alcohol-related crash, alcohol-related fatal crash, traffic accident, impaired driving, driving while intoxicated, driving under the influence, impaired-driving laws, drinking-and-driving laws, law enforcement, roadside sobriety checkpoint, blood alcohol content

## Abstract

Impaired driving remains a significant public health problem in the United States. Although impressive reductions in alcohol-related fatalities occurred between 1982 and 1997, during which all 50 States enacted the basic impaired-driving laws, progress has stagnated over the last decade. Substantial changes in the laws and policies or funding for the enforcement of the criminal offense of driving while intoxicated (DWI) are needed for further substantial progress in reducing alcohol-related crash injuries. However, research indicates that evidence-based laws in the 50 States and current best practices in DWI enforcement are not being fully adopted or used. It seems, however, that effective operations, such as the low-staff check points that are routinely applied in many communities, could be extended to many more police departments. In addition, several enforcement methods have been proposed but never fully tested.

In 1988, the U.S. Surgeon General’s Workshop on Drunk Driving called attention to the broad range of strategies that affect the problem of impaired driving (U.S. Department of Health and Human Services 1989). Research presented at the workshop ranged from the effects of pricing, availability, advertising, marketing, and general epidemiology of alcohol consumption and alcohol problems to safety education, impaired-driving laws, sanctions, and treatment programs for offenders. The proceedings of that workshop demonstrated that impaired driving involves extremely broad public health aspects that cannot be covered in this brief article. Rather, this article reviews only the traditional, century-old measure of deterring vehicle operators from driving while intoxicated (DWI) through laws (see table for a summary of these laws), law enforcement, and public-education programs. A case can be made that general deterrence strategies have had the most immediate and largest impact on the problem.

## The Problem

There are more registered vehicles in the United States than there are licensed drivers to operate them. It is therefore not surprising that in a country where two-thirds of the citizens drink alcohol, impaired driving is a significant public health problem. In the United States, alcohol has been associated with traffic crashes for more than 100 years, as indicated by the publication of the first scientific report on the effect of drinking by operators of “motorized wagons” in 1904 (*Quarterly Journal of Inebriety* 1904). The National Highway Traffic Safety Administration (NHTSA) estimated that impaired driving resulted in more than 14,000 deaths and 500,000 injuries in 2000 and cost society $51 billion in that year (Blincoe et al. 2000). Although alcohol consumption is legal for U.S. citizens aged 21 or older, it is illegal in all States to drive with a blood alcohol concentration (BAC) of 0.08 g/dL or greater (NHTSA 2004). In 2008, there were an estimated 11,773 traffic-crash fatalities involving drivers with BACs of 0.08 g/dL or higher (NHTSA 2009). Each year, more than 1.4 million drivers are arrested for DWI or driving under the influence (DUI) in the United States (Federal Bureau of Investigation 2008).

## Evidence That Laws Addressing Impaired Driving Can Be Effective

**Figure 1:**
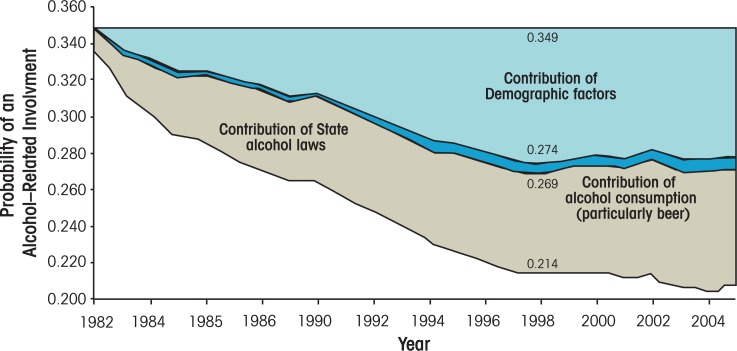
Probability of alcohol involvement of drivers with BACs of .08 or greater in fatal crashes Source: Dang, 2008

**Figure 2:**
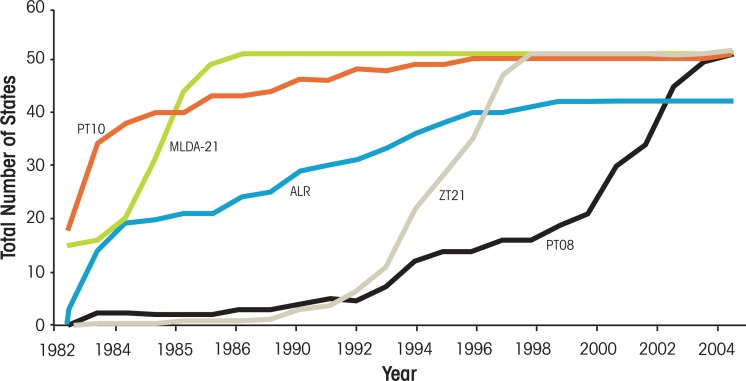
Number of States that enacted alcohol laws (by year and by law). Source: Dang, 2008

From 1983 to 1997, the United States experienced a remarkable reduction in alcohol-related fatal crashes. An analysis of fatal-crash data from 1982 to 2005 estimated that five basic alcohol safety laws accounted for 44 percent of the reduction in fatal crashes, as shown in figure 1 (Dang 2008). This analysis included data from the Fatality Analysis Reporting System (FARS), which collects data on all fatal crashes occurring in the United States and is maintained by the NHTSA, as well as data from several other sources to account for factors such as age, gender, per capita alcohol consumption, time of day and day of the week the crash occurred, presence of important impaired-driving laws, and the level of impaired-driving enforcement that might moderate the influence of BAC on crashes. Figure 1 (upper light-shaded area, labeled “Contribution of demographic factors”) shows the reductions in fatal crashes that Dang’s analysis indicated can be attributed to the aging of the population and the increase in the proportion of female drivers, because female and older drivers have fewer alcohol-related fatal crashes. The dark band [labeled “Contribution of alcohol consumption (particularly beer)”] in figure 1 indicates the small reduction that can be attributed to the minor drop in per capita alcohol consumption over the 24-year study. The lower shaded area (labeled “Contribution of state alcohol laws”) in figure 1 shows the proportion of the total reduction that Dang’s analysis attributed to five basic alcohol safety laws enacted by most of the States during that period. Figure 2 (Dang 2008) shows the trends in the enactment of those five laws: (1) illegal-per-se laws, which make it illegal for a driver to have a BAC of 0.10 g/dL or higher (figure 2, PT10) (Voas et al. 2000; Zador et al. 1988); (2) the law lowering the illegal-per-se level to 0.08 g/dL BAC (figure 2, PT08) (Bernat et al. 2004; Hingson et al. 1996, 2000; Shults et al. 2001; Tippetts et al. 2005; Wagenaar et al. 2007); (3) the administrative license revocation/suspension law that provides for immediate suspension of the driver’s license upon arrest if the offender has a BAC higher than the legal limit (figure 2, ALR) (Wagenaar and MaldonadoMolina 2007; Zador et al. 1988); (4) the minimum legal drinking age (MLDA) law that prohibits individuals younger than age 21 from purchasing or possessing alcohol (figure 2, MLDA-21) (Decker et al. 1988; Fell et al. 2009; O’Malley and Wagenaar 1991; Shults et al. 2001; Toomey et al. 1996; Wagenaar and Toomey 2002); and (5) the zero-tolerance law for drivers younger than age 21 who are not permitted to have any alcohol in their systems while driving (figure 2, ZT21) (Hingson et al. 1992, 1994; Voas et al. 2003).

Dang estimates that these alcohol safety laws affect drivers at all BAC levels, including those with BACs lower than the 0.08 g/dL legal limit and those with BACs of 0.20 g/dL or greater. She notes that during the last decade of the study, there was no real change in alcohol-related fatalities and suggests that the alcohol safety laws, although not producing additional reductions, continue to be effective in maintaining the lower rate of alcohol involvement in fatal crashes.

**Figure 3:**
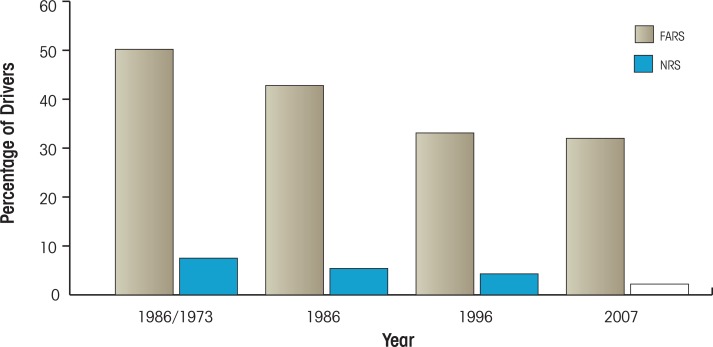
Percentage of drivers on the road on weekend evenings with BACs ≥0.08 g/dL, as determined by the National Roadside Surveys (NRS), versus percentage of drivers killed in fatal crashes with BACs ≥0.08 g/dL, as determined by the Fatality Analysis Reporting System (FARS) 1973 to 2007. Source: Lacey, et al., 2009

The National Roadside Surveys (NRSs), which have collected breath tests from a U.S. representative sample of weekend nighttime drivers every decade beginning in the 1970s, provide some evidence that lower rates of drinking and driving may be continuing (Lacey et al. 2009). Figure 3 shows the decline in the percentage of fatally injured drivers with BACs of 0.08 g/dL or greater over the last four decades compared with the decline in the percentage of drivers on the roads with those illegal BACs, according to the NRSs. Although the impaired-driving fatal-crash rate does not seem to have been reduced during the last decade (1996–2007), the percentage of nighttime, weekend drivers with BACs of 0.08 g/dL or higher seems to have declined (from 4.3 to 2.2 percent). However, this decline of impaired drivers on U.S. roads needs to be interpreted cautiously because the number of drivers refusing to participate in the 2007 survey was greater than in previous NRS studies.

## Development of Chemical Tests for Alcohol

The decrease in impaired-driving crashes in the United States coincided with similar trends in other industrialized nations (Sweedler et al. 2004). The reductions experienced during the last one-third of the 20th century by many countries may have been attributed, at least in part, to the adoption of scientific advances in the methods for measuring BACs and the use of that information in programs to reduce impaired driving. When impaired driving was first criminalized early in the 20th century (i.e., New York passed the first law in 1910), the offense was defined as “driving while intoxicated” or “driving under the influence.” Police officers were required to record the arrested driver’s behavior and then testify in court using these relatively vague terms. Widmark’s work (1932) in relating alcohol consumption to BAC and the development of relatively simple but accurate methods for measuring BAC (Jones 2000) during the half-century after the criminalization of impaired driving provided the scientific basis for a more rigorous definition of impaired driving.

**Figure 4:**
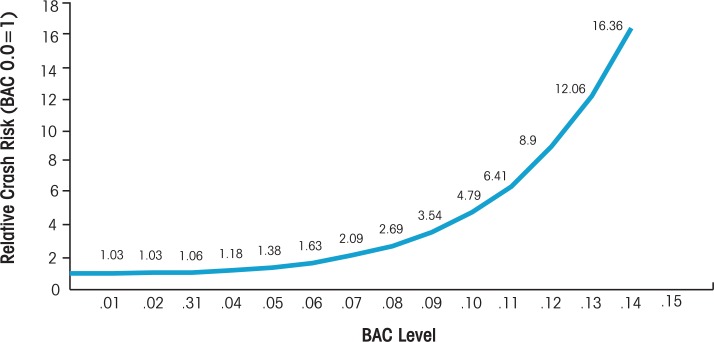
Relative risk of crash involvement as a function of driver’s BAC. Source: Accepted from Blomberg et al. 2009.

BACs can be determined with substantial precision compared with the relatively subjective behavioral signs of intoxication. In addition, it provides a means of quantifying the relative risk of crash involvement as a function of alcohol consumption as measured in BAC units. That relationship is shown in figure 4, which illustrates the rise in crash risk as BAC increases on the basis of a case–control study by Blomberg and colleagues (2009). In that study, BAC data were collected for crash-involved drivers and paired with non–crash-involved drivers using the roads at the same location and at the same time of day and day of week as the crash-involved drivers. Such relative-risk studies have encouraged the adoption of illegal-per-se laws by national legislatures in European countries and by State and provincial legislatures in the United States, Canada, and Australia. Laws based on the BAC level increase the efficiency of enforcement efforts. For example, in the past, Swedish laws required that a physician examine a driver accused of impaired driving before the charge could be brought, but with the per se law, it became possible to proceed on the basis of a blood test. The use of BAC measures in the criminal-justice system also stimulated the development of breath tests for BACs, such as the Borkenstein and Smith’s (1961) breathalyzer, which allowed ordinary police officers to conduct evidential quality tests at local police stations.

This process culminated in the 1960s and 1970s with the development of accurate handheld preliminary breath test (PBT) units that officers could use in the field. This resulted in a revolution in the methods for enforcing impaired-driving laws through the combination of field BAC tests and the random stopping of motorists. Australia carried this process the furthest by developing a random-breath-testing (RBT) enforcement system in which officers were stopping motorists at random (day and night) and administering a mandatory PBT. A reading higher than the 0.05 g/dL BAC limit for driving resulted in additional onsite testing or immediate transportation of the driver to the police station for evidential BAC testing (Cameron et al. 1997; Homel 1988). This procedure could be classified as a “chemistry-based” system, because conceptually, the driver’s behavior does not play a role in the arrest process (Voas and Lacey 1990). RBT is believed to derive its power from the uncertainty it creates in potential DWI offenders about their ability to avoid attracting the attention of the police by driving slowly and carefully. It also counters the notion that, if stopped, the offender can avoid appearing impaired and therefore will not be required to take a BAC test. Sweden adopted the RBT procedure, but other nations, such as Great Britain, adopted more limited versions of the field-testing program that did not provide for random stopping but allowed police to require a field breath test under specified conditions, such as involvement in a crash or arrest for a traffic offense (Ross 1984).

## Constitutional Limits on the Application of RBT and Illegal-Per-Se Laws in the United States

In the United States, the use of per se laws, BAC technology, and random stopping has been limited by the Fourth Amendment to the U.S. Constitution, which requires that searches and seizures be reasonable. The U.S. Supreme Court in *Michigan Department of State Police v. Sitz* (1990) held that the State’s duty to protect citizens from impaired drivers outweighed the small intrusion involved in the brief stopping of a vehicle without individual suspicion at sobriety checkpoints. Conducting a breath test (a search) with a handheld unit, which requires less than 30 seconds, also might have been considered a minor intrusion because it could have been justified by the need of the State to protect the driving public. This possibility has never been reviewed by the Supreme Court, however. As a result, in the United States, roadside breath tests cannot be required without a basis for suspecting that the driver is impaired. Thus, RBT in the United States is limited to stopping motorists at specially designed checkpoints, conducting brief interrogations, and observing the drivers to determine whether they are under the influence of alcohol. Only when the signs of impairment are present can an officer test the driver for BAC.

The failure to find a compromise on the Fourth Amendment prohibition against random preliminary breath testing in the field was exacerbated by State policies regarding the evidential chemical testing of breath or blood once an offender is arrested. Although the U.S. Supreme Court in *Schmerber v. State of California* (86 S Ct 1826 [1966]) held that drivers validly arrested for DWI had no right to refuse a BAC test, the States enacted implied-consent laws to avoid the possibility of having to restrain offenders to force a BAC test. Such laws allowed drivers charged with DWI to refuse the BAC test but at the cost of having their driver’s licenses suspended. Given the heavy penalties associated with a DWI conviction, a limited period of license suspension has not proved to be a sufficient motivation to ensure the acceptance of the evidential test. Chemical-test refusal rates run as high as 71 percent in some States (Voas et al. 2009), and test refusals are a significant problem in enforcing DWI laws for both the police and prosecutors (Robertson and Simpson 2002; Simpson and Robertson 2001).

The effectiveness of the State BAC per se laws, aside from being compromised by the ability of DWI offenders to avoid chemical testing, faces another limitation: the test only can be required if there is probable cause to make a DWI arrest. This leaves an opening for the defense attorney to challenge the officer’s report on the driver’s behavior at the roadside and demonstrate that there was insufficient evidence to make the arrest, in which case the BAC test is inadmissible. Therefore, States must maintain their historic impaired-driving laws on the basis of driver behavior because the requirement to take a BAC test rests on probable cause to make the arrest. These problems and policies developed in reaction to Fourth Amendment requirements significantly limit the extent to which the illegal-per-se concept can be applied in the United States. This has led to a hybrid enforcement system described below.

## The U.S. DWI Enforcement System

Aside from new breath-test technologies, two factors played a major role in the reduction of impaired-driving fatal crashes described above and shown in figure 1. First, the establishment of the Department of Transportation and its highway-safety arm, NHTSA in 1966, and the enactment of the Motor Vehicle and Highway Safety Acts provided funds and technical support for DWI enforcement. Second, the emergence of citizen activism in 1980, led by Mothers Against Drunk Driving (MADD), helped focus public attention on drunken driving and stimulated support for enhanced impaired-driving laws (Fell and Voas 2006).

The growth of the citizens’ activist movement was associated with a substantial increase in media coverage of drinking and driving and a fivefold increase in the number of drinking-and-driving bills in State legislatures (Howland 1988). MADD established offices and chapters in the majority of States and provided an active legislative education program that promoted the passage of the evidence-based DWI laws enacted during the last 30 years.

The current U.S. effort to deter impaired driving falls roughly into two areas: enforcing existing impaired-driving laws and publicizing enforcement activities to ensure public awareness. Because deterrence is based on the perception of the probability of apprehension and sanctioning and not on the actual numbers of citations and sanction actions (Ross 1984), emphasis has been placed on high-visibility enforcement that attracts public attention. There currently are three broad classes of DWI enforcement: (1) standard traffic enforcement operations, in which officers stop vehicles to issue traffic citations for aberrant driving and, if alerted to the possibility that a driver is impaired, initiate a DWI investigation and make an arrest if appropriate; (2) dedicated patrol operations on weekend nights, where officers concentrate on the detection of DWI offenders (these dedicated patrol procedures substantially increase DWI arrests because the most highly skilled and motivated officers are assigned to these operations and they are mounted at times when impaired drivers are most prevalent on the roadways); and (3) sobriety checkpoints. Both dedicated patrols and sobriety checkpoints are viewed as high-visibility methods because they are visible to the driving public, attract media attention, and therefore can be publicized in print and electronic media. However, both of these high-visibility enforcement strategies often depend on Federal funding from the State Highway Safety Offices rather than the local enforcement budget. This means that many, if not most, police departments limit the use of both of these methods.

Despite the inability to use breath testing as a detection method in the United States, sobriety checkpoints have proven to be more effective than the dedicated patrol operations involving officers searching for impaired drivers (Stuster and Blowers 1995). Moreover, Elder and colleagues (2002) conducted a meta-analysis of 10 U.S. sobriety checkpoint studies and found a mean reduction of 20 percent in crashes likely to have involved alcohol. It is interesting to note that this reduction actually was slightly higher than the 18 percent crash reduction documented through a meta-analyses of RBT programs in Australia (Shults et al. 2001), suggesting that, despite their relative inefficiency, U.S sobriety checkpoints are having an effect similar to that of RBT in Australia.

Given this evidence for their effectiveness, checkpoints have not been as widely applied in the United States, as might have been expected. Aside from the 12 States that do not permit checkpoints under their constitutions or prohibit them for some other reason (State statute), only 24 States and the District of Columbia conduct checkpoints as frequently as once a month. In the remaining 14 States, checkpoints only are implemented on special holidays, such as the Fourth of July or New Year’s Eve (Fell et al. 2003), if conducted at all. Police departments resist using checkpoints because they produce relatively few DWI arrests and are believed to require a large staff of officers, making them expensive. Despite the research cited herein, which supports the effectiveness of checkpoints even though they produce few DWI arrests, most police departments remain unconvinced that checkpoints are cost-effective. At least one study (Miller et al. 1998), however, indicates that sobriety checkpoints are very cost-effective. Moreover, to overcome this objection, the NHTSA and the Insurance Institute for Highway Safety have funded studies that use a small number of officers (four to six) to conduct checkpoints, demonstrating that such low-staff operations can be cost-effective (Lacey et al. 2006; Stuster and Blowers 1995).

The low DWI arrest rates at checkpoints result principally from the inability to breath test all the drivers being interviewed. Research on breath tests of drivers who have passed through checkpoints indicate that this limitation to observing and questioning drivers, rather than testing each driver’s BAC as is done in RBT programs, results in police missing one-half of those who are over the BAC limit (Ferguson et al. 1995; Jones and Lund 1986; Wells et al. 1997). This limitation also may affect the deterrent value of checkpoints, as indicated by Beck and Moser (2006), who surveyed drivers passing through checkpoints and compared them with drivers passing by a checkpoint. Drivers experiencing a checkpoint interview reported less concern about being arrested at a checkpoint than drivers merely passing by a checkpoint.

A partial technological solution to this problem has been the development of a passive alcohol sensor (PAS). This unit can be mounted in the officer’s flashlight and can draw in mixed expired air from 4 to 6 inches in front of the driver’s face. These PAS devices provide an indication of the individual’s probable BAC (Voas et al. 2006). Although not reviewed by the U.S. Supreme Court, arrests in which the PAS plays a role generally have been accepted by the courts as constitutional (Fields and Henricko 1986; Manak 1986). Studies have indicated that when passive-sensing flashlights are used at checkpoints, about one-half of the over-the-limit drivers who were not initially detected by the police interview are identified by officers using the PAS (e.g., Ferguson et al. 1995). Thus, research suggests that both of the key concerns with checkpoints expressed by police could be overcome. It is unfortunate that this information has not yet influenced policy in most police jurisdictions in the United States.

Because of these limitations, much of the research funded by the NHTSA has focused on educating officers to observe a driver’s behavior objectively in each of the three stages of the arrest process: (1) identifying vehicles driven by impaired drivers (e.g., weaving in the lane) (Stuster 1997); (2) detecting driver impairment through an interview with the driver at the roadside during the traffic stop (i.e., observing fumbling with keys, slurred speech) (Stuster 1997); and (3) conducting standardized field sobriety tests (SFSTs) once there is evidence to invite the driver out of the vehicle. The three tests in the SFST are the one-legged stand, the walk and turn, and the horizontal gaze nystagmus (HGN). The SFST, originally developed by Tharp and colleagues (1981), has proven to be generally successful in objectifying the officers’ estimates of driver impairment. NHTSA has substantially funded Nationwide training programs for police officers on SFST procedures. As a result, SFSTs have become the key element in the arrest process, providing the basis for requiring an evidential BAC test, and, in the absence of a BAC test, the most important evidence on which to obtain a conviction. However, some State courts do not allow the introduction of evidence from the HGN test in DWI trials, and it is the best predictor of the three SFSTs.

## Public Information Programs

Because the deterrence theory is based on the concept that the perceived risk of detection and sanctioning is of central importance, publicity plays a key role in law enforcement. News coverage of an ongoing enforcement program arguably is the most powerful media tool for creating deterrence (Atkin 1989, p. 22). However, the number of studies in which the role of the news media has been measured is very limited. Voas and Hause (1987) found that strong news coverage doubled the effect of an intensive enforcement program on alcohol-related crashes. In another study, Voas and colleagues (1997) measured print and electronic news media associated with an intensive DWI-enforcement program and found that media activity was associated with (1) the increased perceived risk of arrest by the public, (2) decreased prevalence of drinking drivers on the road, and (3) a reduction in alcohol-related crashes. Media advocacy (Wallack and Sciandra 1990–1991) methods can be used to attract news coverage of enforcement programs by conducting news conferences and designing media events related to the enforcement operations.

Over the last half-century, public service announcements, produced at no cost to the government and broadcast free by media companies, have been a ubiquitous feature of national impaired-driving campaigns. Their efficacy, however, remains essentially unmeasured (Atkin 1989). Since 2002, there has been increased interest in using paid media, and the U.S. Congress has provided funds (approximately $20 million annually) to conduct two national media and enforcement campaigns directed at impaired driving during the Labor Day and Christmas/New Year’s periods. These mobilizations have had limited evaluations but hold promise in that they seem to at least keep impaired driving at its current low level (NHTSA 2007*b*).

## Community Programs

Community programs, in which an organization or coalition is established to support DWI enforcement, have become very popular. The NHTSA has provided manuals for establishing such organizations/coalitions and describing the role they can play in supporting DWI-enforcement programs. The first examples of such organizations were the Alcohol Safety Action Projects (ASAPs), funded in 35 communities by the NHTSA in the 1970s. Those projects provided funding for enforcement, public information, adjudication, and treatment programs directed at impaired driving. Evaluation of the ASAPs suggested that those that increased DWI enforcement were successful in reducing alcohol-related crashes. Three other prominent community programs principally directed at impaired driving have produced alcohol-related crash reductions (Hingson et al. 1996, 2005; Holder et al. 2000). The evidence on Statewide impaired-driving enforcement programs is mixed. A checkpoint program in Tennessee was highly successful, resulting in a 20 percent reduction in impaired-driving fatalities in the first year of implementation (Lacey et al. 1999). More recently, evaluations of highly publicized Statewide enforcement-demonstration projects showed desired effects in Georgia, Indiana, Michigan, and Tennessee but not in Louisiana, Pennsylvania, or Texas, (Fell et al. 2008).

## Potential Future Developments in Enforcement Programs

The lack of substantial progress during the last 15 years in reducing the involvement of impaired drivers in fatal crashes (see figure 1) has stimulated interest in identifying policies that have potential for lowering the rate of alcohol-related crashes. Several policies have been evaluated and found effective but are not currently being implemented. Other potential laws and countermeasures also are candidates for evaluation and, if found to be effective, should be implemented. Among these are the following:

- Increase the use of low-staff sobriety checkpoints. Because police departments view checkpoints as expensive operations that require paying overtime for the assigned officers, there is an incentive for persuading police-department officials to use low-staff checkpoints that can be mounted within the normal staffing of weekend patrols. As noted, low-staff checkpoints have been demonstrated to be effective (Lacey et al. 2006; Stuster and Blowers 1995; Voas 2008; Voas et al. 1985). The untested “PASpoint” checkpoint system (Voas et al. 2005), featuring short periods of checkpoint operations using passive sensors interspersed with a regular patrol operation, could be implemented by small- to medium-sized police departments. Increasing the use of passive-sensing flashlights at checkpoints should increase the number of DWI offenders detected at such operations by 50 percent. In addition, publicizing the use of such units may increase the public’s perception that if they drive after drinking, they are more likely to be apprehended. Thus, the use of passive sensors also could serve as a general deterrent.
- Reduce the current 0.08 g/dL BAC limit to 0.05 g/dL.Strong evidence indicates that reducing the legal BAC limit from 0.10 g/dL to 0.08 g/dL reduced impaired-driving fatalities (Tippetts et al. 2005; Wagenaar et al. 2007). Therefore, if the United States lowers the BAC limit to the 0.05 g/dL level established in Australia, New Zealand, and the countries in the European Economic Union, an additional reduction in alcohol-related fatalities should be expected. Evidence for this expectation includes the following: when the limit was reduced from0.08 g/dL to 0.05 g/dL in Queensland, Australia, alcohol-related fatal crashes on Saturdays were reduced by 18 percent (Henstridge et al. 1995); when the legal BAC limit was lowered from 0.10 g/dL to 0.08 g/dL in the United States, crashes involving drivers at all BAC levels were reduced, not just those in the affected 0.08 g/dL and 0.09 g/dL interval (Dang 2008); Moskowitz and colleagues (2000) reviewed the literature on laboratory, simulator, and closed-course driving on driver performance and found that impaired performance could be documented at 0.05 g/dL and lower BACs, down to 0.02; a study by Blomberg and colleagues (2009) (shown in figure 4) found a significant 37 percent increase in crash risk at the 0.05 g/dL BAC level. Fell and Voas (2009) have summarized the evidence for lowering the limit to 0.05 g/dL.
- Increase the number of national public media mobilizations directed at increased DWI enforcement from the two current campaigns to four mobilizations: (i.e., one for each quarter). There is evidence that national mobilizations increase local enforcement activity and reduce crashes (NHTSA 2007*a*, *b*; Solomon et al. 2002).
- Enhance the prestige of DWI enforcement by creating a specialized training program and status recognition for officers specializing in impaired-driving enforcement. The Drug Recognition Expert Program, developed to provide officers with training to identify the impairment produced by different substances, has proved to be very popular with police officers and has attracted the most qualified young officers to take part in the program. It is common that one or two officers within a department will take a special interest in DWI enforcement and arrest several times the number of offenders apprehended by other officers in the same enforcement squad. For example, it may be possible to make DWI enforcement an elite activity in which the officers specially trained in the use of the latest equipment and enforcement procedures would be recognized with special badges, bonuses, and vehicles with special insignias.
- Repeal implied-consent laws, thereby requiring BAC testing of all arrested drivers, by force if necessary. Research has demonstrated that police departments can be staffed with phlebotomists, and BAC tests can be administered to all DWI arrestees (Lacey et al. 2003). Though this procedure has not yet been demonstrated to reduce alcohol-related crashes, there is supporting evidence that avoiding the BAC test reduces the probability of conviction and that those who refuse have higher DWI recidivism rates (Voas et al. 2009).
- Criminalize refusal of the BAC test by making the sanctions equivalent to conviction for DWI. This is essentially the system that has been effective in minimizing refusals in Australia and Europe. Several U.S. States have criminalized chemical test refusal, but this procedure has not been fully evaluated (Voas et al. 2009).
- Reconsider using PBT in the field. It is possible that the U.S. Supreme Court, acting within the context of decisions related to the random stopping of motorists (Michigan Department of State Police v. Sitz, 1990) and within the new social context of airline security, will find that the intrusion presented by the PBT is sufficiently modest and the need of the State to protect its citizens against drunk drivers is sufficiently strong to justify laws requiring motorists to submit to such tests at checkpoints and perhaps in other enforcement operations.

## Summary

Impaired driving remains a significant public health problem in the United States. Impressive reductions in alcohol-related fatalities occurred from 1982 to 1997, during which all 50 States enacted the basic impaired-driving laws. Although the current enforcement system is preserving those substantial benefits, progress has stagnated over the last decade. There is little indication that, without substantial changes in the laws and policies or funding for DWI enforcement, there will be further substantial progress in reducing alcohol-related crash injuries. However, research indicates that evidence-based laws in the 50 States and current best practices in DWI enforcement are not being fully adopted or used. Some of these strategies may simply not be politically feasible. It would seem, however, that effective operations, such as low-staff checkpoints that are routinely applied in many communities, could be extended to many more police departments. In addition, several enforcement methods have been proposed but never fully tested. These offer opportunities for future enforcement research. Finally, it is important to remember that impaired driving is influenced by a broad set of environmental and policy variables not directly related to impaired-driving laws. These variables, which are discussed in other articles in this special issue of *Alcohol Research & Health,* can have significant effects on alcohol-related highway injuries and deaths.

## Figures and Tables

**Table:** Impaired-Driving Legislation and Policies in the United States

**State**	**BAC Per Se Level**	**ALR**	**Vehicle Sanctions for Repeat DWI**	**More Serious Penalities for High BAC**	**GDL**	**Primary Belt Law**	**Sobriety Checkpoints**	**Interlocks for All Convicted Offenders**
Alabama	.08	Yes	Yes	No	Yes	Yes	Yes	No
Alaska	.08	Yes	Yes	Yes	Yes	Yes	No	Yes
Arizona	.08	Yes	Yes	Yes	Yes	No	Yes	Yes
Arkansas	.08	Yes	Yes	Yes	Yes	Yes	Yes	Yes
California	.08	Yes	Yes	Yes	Yes	Yes	Yes	No
Colorado	.08	Yes	Yes	Yes	Yes	No	Yes	Yes
Connecticut	.08	Yes	Yes	Yes	Yes	Yes	Yes	No
Delaware	.08	Yes	Yes	Yes	Yes	Yes	Yes	No
Dist. of Col.	.08	Yes	Yes	Yes	Yes	Yes	Yes	No
Florida	.08	Yes	Yes	Yes	Yes	No	Yes	No
Georgia	.08	Yes	Yes	Yes	Yes	Yes	Yes	No
Hawaii	.08	Yes	Yes	Yes	Yes	Yes	Yes	No
Idaho	.08	Yes	Yes	Yes	Yes	No	No	No
Illinois	.08	Yes	Yes	Yes	Yes	Yes	Yes	Yes
Indiana	.08	Yes	Yes	Yes	Yes	Yes	Yes	No
Iowa	.08	Yes	Yes	Yes	Yes	Yes	No	No
Kansas	.08	Yes	Yes	Yes	Yes	No	Yes	No
Kentucky	.08	No	Yes	Yes	Yes	Yes	Yes	No
Louisiana	.08	Yes	Yes	Yes	Yes	Yes	Yes	Yes
Maine	.08	Yes	Yes	Yes	Yes	Yes	Yes	No
Maryland	.08	Yes	Yes	No	Yes	Yes	Yes	No
Massachusetts	.08	Yes	Yes	Yes	Yes	No	Yes	No
Michigan	.08	No	Yes	No	Yes	Yes	No	No
Minnesota	.08	Yes	Yes	Yes	Yes	No	No	No
Mississippi	.08	Yes	Yes	No	Yes	Yes	Yes	No
Missouri	.08	Yes	Yes	Yes	Yes	No	Yes	No
Montana	.08	No	Yes	Yes	Yes	No	No	No
Nebraska	.08	Yes	Yes	Yes	Yes	No	Yes	Yes
Nevada	.08	Yes	Yes	Yes	Yes	No	Yes	No
New Hamp.	.08	Yes	Yes	Yes	Yes	No	Yes	No
New Jersey	.08	No	Yes	No	Yes	Yes	Yes	No
New Mexico	.08	Yes	Yes	Yes	Yes	Yes	Yes	Yes
New York	.08	No	Yes	Yes	Yes	Yes	Yes	No
N. Carolina	.08	Yes	Yes	Yes	Yes	Yes	Yes	No
N. Dakota	.08	Yes	Yes	Yes	No	No	Yes	No
Ohio	.08	Yes	Yes	Yes	Yes	No	Yes	No
Oklahoma	.08	Yes	Yes	Yes	Yes	Yes	Yes	No
Oregon	.08	Yes	Yes	No	Yes	Yes	No	No
Pennsylvania	.08	No	Yes	Yes	Yes	No	Yes	No
Rhode Island	.08	No	Yes	Yes	Yes	No	No	No
S. Carolina	.08	Yes	Yes	Yes	Yes	Yes	Yes	No
S. Dakota	.08	No	Yes	Yes	Yes	No	Yes	No
Tennessee	.08	No	Yes	Yes	Yes	Yes	Yes	No
Texas	.08	Yes	Yes	Yes	Yes	Yes	No	No
Utah	.08	Yes	Yes	Yes	Yes	No	Yes	No
Vermont	.08	Yes	Yes	No	Yes	No	Yes	No
Virginia	.08	Yes	Yes	Yes	Yes	No	Yes	No
Washington	.08	Yes	Yes	Yes	Yes	Yes	No	Yes
W. Virginia	.08	Yes	Yes	No	Yes	No	Yes	No
Wisconsin	.08	Yes	Yes	Yes	Yes	No	No	No
Wyoming	.08	Yes	Yes	No	Yes	No	No	No
**National Totals**	**50 States plus DC**	**41 States plus DC**	**50 States plus DC**	**41 States plus DC**	**49 States plus DC**	**27 States plus DC**	**38 States plus DC**	**9 States**

NOTE: ALR = administrative license revokation; GDL = graduated driver licensing.
